# uDISE model: a universal drug-induced sedation endoscopy classification system—part 1

**DOI:** 10.1007/s00405-017-4597-5

**Published:** 2017-05-10

**Authors:** Esuabom Dijemeni, Gabriele D’Amone, Israel Gbati

**Affiliations:** 1 Department of Bioengineering, Imperial College London, Royal School of Mines, Prince Consort Road, London, SW7 2BP UK; 2 Research and Development, DISE INNOVATION, London, UK; 3 Dyson School of Design Engineering, Imperial College London, London, UK; 4 School of Design, Royal College of Art, London, UK; 5 Department of Mechanical Engineering, Imperial College London, London, UK

**Keywords:** Drug-induced sedation endoscopy, Classification, Sleep apnea, Upper airway obstruction, Sleep

## Abstract

Drug-induced sedation endoscopy (DISE) classification systems play a significant role in clinical analysis based on DISE findings, treatment decision process, treatment planning process and fundamentally in treatment outcomes. However, there is a major problem: there is no universally agreed DISE classification system. Hence, for the same DISE examination different DISE classification systems can be used to: assess anatomic findings, decide and plan different treatments. Hence, this leads to different treatment outcomes. The key objective of this study is to propose uDISE model: universal drug-induced sedation endoscopy (DISE) classification system. Set theory and relational mapping was used to develop a DISE classification system based on anatomical structures/level; degree of severity; and configuration of obstruction and its relationship with existing DISE classification systems. uDISE model consists of seven anatomical sites (nose, velum, tonsils, lateral pharyngeal wall/oropharynx, tongue base, epiglottis and larynx), three degrees of obstructive severity (none, partial and complete), three configurations of obstruction (anteroposterior, lateral and circumferential) and a severity index. uDISE model was mapped to four existing DISE classification systems: Pringle and Croft grading system, VOTE, NOHL and P-T-L-Tb-E. uDISE model provides a methodology for mapping different DISE findings based on different classification systems into one common DISE assessments format. This provides a framework for comparing different DISE assessments, treatment plan and treatment outcome irrespective of DISE classification system used. Further research is required to establish a complete relational mapping between uDISE model and other existing DISE classification systems.

## Introduction

When surgery is the considered treatment intervention for obstructive sleep apnea, identification, analysis and reporting of upper airway obstruction during sleep is important for optimal surgical intervention outcomes [1]. Croft and Pringle proposed drug-induced sleep endoscopy (DISE) as an endoscopic evaluation of the dynamic changes of the upper airway during sedated sleep [2].

DISE has been shown to:

1. Provide 3D dynamic visualisation of multi-segmental upper airway obstruction [3].
2. Provide quality assessment of dynamic airway events [4];
3. Provide useful information on patient management [5].
4. Improve treatment planning as compared to using awake upper airway assessments technique [6].
5. Have reliable intraobserver agreement [7].
6. Have moderate to substantial interrater reliability [8].
7. Have good test–rest reliability [9].
8. Have good agreement with polysomnography [10].

Some criticisms of DISE includes:

1. Changes in snoring patterns in sedated sleep as compared to natural sleep [11].
2. Merely a snapshot of snoring; not representative of how snoring changes throughout the sleep [12].
3. Subjective evaluation assessment [13].
4. A growing number for multiple classification systems [14].

However, DISE is growing to become the gold standard for observing dynamic upper airway obstruction during sleep.

This study focuses on the problem of multiple DISE classification systems. At the first European Position Meeting on DISE, the problem was stated “It is disappointing that so far no consensus has been reached on a scoring and classification system… The goal remains to come to one universally accepted classification system” [14]. Furthermore, seven different DISE classification systems were highlighted in the European position paper on DISE [14] (for more details on different DISE classification system, readers should read Table 3 in [14] and references [2, 7, 15–30]). Furthermore, it has been suggested that there are potentially over 16 DISE classification system proposed in the literature [31].

The objectives of this study are to:

1. Develop a universal DISE classification system, uDISE model.
2. Provide a framework for relating uDISE model with other existing DISE classification systems.

## DISE classification system

## Methods

### uDISE model

uDISE model classification system was developed by analysing 18 existing DISE classification systems based on anatomical structures, anatomical levels, severity of obstruction and configuration of obstruction [2, 7, 15–30].

#### Classification referential mapping

uDISE model was mapped to four existing DISE classification systems using set theory and relational mapping based on anatomical structures, anatomical levels, severity of obstruction and configuration of obstruction.

## Results

Table 1 uDISE model.

**Table 1:** uDISE model

Structures	Degree of obstruction^a^	Configuration
Nose	No obstruction	Anteroposterior
Velum		
Tonsils	Partial obstruction	Lateral
Lateral pharyngeal wall		
Tongue base	Complete obstruction	Concentric
Epiglottis		
Larynx		

*AP* anteroposterior, *L* lateral, *C* concentric

^a^0: no obstruction (no vibration, <50%); 1: partial obstruction (vibration 50–75%), 2: complete obstruction (collapse, >75%)

Table 2 referential classification table.

**Table 2:** Referential classification table

uDISE	Pringle [15]	Kezirian [21]	Vinci [22]	P-T-LTb-E [29]
N_0_	X	X	N_v 1–2_	X
N_1_	X	X	N_v 3_	X
N_2_	X	X	N_v 4_	X
V_0 AP_	G1_P_	V_k 0 AP_	O_V 1–2 T_	P_V 1_
V_1 AP_	G1_P_	V_k 1 AP_	O_V 3 T_	P_V 2_
V_2 AP_	G2_P_	V_k 2 AP_	O_V 4 T_	P_V 2_
V_0 L_	G1_P_	V_k 0 L_	O_V 1–2 L_	–
V_1 L_	G1_P_	V_k 1 L_	O_V 3 L_	–
V_2 L_	G2_P_	V_k 2 L_	O_V 4 L_	–
V_0 C_	G1_P_	V_k 0 C_	O_V 1–2 C_	P_V 1_
V_1 C_	G1_P_	V_k 1 C_	O_V 3 C_	P_V 3_
V_2 C_	G2_P_	V_k 2 C_	O_V 4 C_	P_V 3_
Ts_0 L_	G1_P_	O_k 0 L_	–	T_V 1_
Ts_1 L_	G1_P_	O_k 1 L_	–	T_V 2_
Ts_2 L_	G2_P_	O_k 2 L_	TS_V 3, 4 T_	T_V 3_
O_0 AP_	–	–	O_V 1–2 T_	–
O_1 AP_	–	–	O_V 3 T_	–
O_2 AP_	G3_+P_, G4_+P_	–	O_V 4 T_	–
O_0 L_	–	O_k 0 L_	O_V 1–2 L_	L_V 1_
O_1 L_	–	O_k 1 L_	O_V 3 L_	L_V 2_
O_2 L_	G3_+P_, G4_+P_	O_k 2 L_	O_V 4 L_	L_V 3_
O_0 C_	–	–	O_V 1–2 C_	–
O_1 C_	–	–	O_V 3 C_	–
O_2 C_	G3_+P_, G4_+P_	–	O_V 4 C_	–
Tb_0 AP_	G5_P_	T_k 0 AP_	H_V 1–2 T_	Tb_V 1_
Tb_1 AP_	G5_P_	T_k 1 AP_	H_V 3 T_	Tb_V 2_
Tb_2 AP_	G5_P_	T_k 2 AP_	H_V 4 T_	Tb_V 3_
Tb_0 L_	G5_P_	–	H_V 1–2 L_	–
Tb_1 L_	G5_P_	–	H_V 3 L_	–
Tb_2 L_	G5_P_	–	H_V 4 L_	–
Tb_0 C_	G5_P_	–	H_V 1–2 C_	–
Tb_1 C_	G5_P_	–	H_V 3 C_	–
Tb_2 C_	G5_P_	–	H_V 4 C_	–
E_0 AP_	G5_P_	E_k 0 AP_	L_N_	E_V 1_
E_1 AP_	G5_P_	E_k 1 AP_	L_P_	E_V 2_
E_2 AP_	G5_P_	E_k 2 AP_	L_P_	E_V 2_
E_0 L_	G5_P_	E_k 0 L_	L_N_	–
E_1 L_	G5_P_	E_k 1 L_	L_P_	–
E_2 L_	G5_P_	E_k 2 L_	L_P_	–
L_0_	X	X	L_N_	X
L_1_	X	X	L_P_	X
L_2_	X	X	L_P_	X

X, site of obstruction not considered anatomically; –, obstruction configuration not considered

## Discussion

### uDISE model

The uDISE model (Table 1) encompasses the most commonly involved structures, encompassing the degree and configuration of obstruction.

#### Severity of obstruction

The severity of obstruction for assessing the degree of airway narrowing is [32]:

1. 0: no obstruction (no vibration, <50%).
2. 1: partial obstruction (vibration 50–75%).
3. 2: complete obstruction (collapse, >75%).

#### Configuration of obstruction

The configuration of obstruction can be described as anteroposterior (anterior structures moving posteriorly against the posterior pharyngeal wall), lateral (structures move laterally towards the centre of the airway) or concentric (combination of the former) [14].

#### Anatomical structures

The seven most common obstruction sites are evaluated in uDISE model: nose, velum, tonsil, oropharynx (lateral walls), tongue base, epiglottis and larynx.

Nasal obstruction was considered to be obstruction in the nasal cavity and nasopharynx [22]. Obstruction at the velum was related to obstruction to the soft palate, uvula or lateral pharyngeal wall tissue at the level of the velopharynx [21]. Due to the indistinguishability of the three structures both anatomically and during DISE examination, they were grouped as velum obstruction [21]. Velum obstruction was considered to occur in the anteroposterior, lateral and concentric configuration. Obstruction at the oropharynx walls was solely based on obstruction involving only lateral pharyngeal wall tissue [21]. Tonsils obstruction was considered as a distinct structure separate from the lateral pharyngeal wall tissue [14, 21, 29]. Both tonsils and lateral pharyngeal wall tissue all collapsed in a lateral configuration [21]. Tongue base obstruction was the narrowing of the airway at the tongue based in the anteroposterior, lateral and circumferential configuration based on the space between the anterior commissure and the posterior pharyngeal wall [7, 21, 29]. Epiglottis obstruction occurred when the epiglottis collapse was either as a result of the anteroposterior prolapse of the folding of the epiglottis with decreased structural rigidity or posterior displacement of the entire epiglottis towards the pharyngeal wall with normal structural integrity or a lateral folding of the epiglottis. Laryngeal obstructions were considered to be obstruction at the supraglottic and glottis [22].

#### Obstruction scoring

An example of a completely normal result would be N_0_V_0_Ts_0_O_0_Tb_0_E_0_L_0_. A tonsillar enlargement causing obstruction will be scored as N_0_V_0_Ts_1L_O_0_Tb_0_E_0_L_0_. A simple palate snoring will be scored as N_0_V_1AP_Ts_0_O_0_Tb_0_E_0_L_0_. A multilevel obstruction with severe tonsillar obstruction and severe tongue based obstruction will score N_0_V_0_Ts_2L_O_0_Tb_2AP_E_0_L_0_.

#### Treatment planning

Ongoing research is examining how uDISE model score relates with surgical treatment [1]. Example of uDISE model score—treatment relationship will be

- N_0_V_1AP_Ts_0_O_0_Tb_0_E_0_L_0_—uvulopalatopharyngoplasty.
- N_0_V_0_Ts_2L_O_0_Tb_2AP_E_0_L_0_—tonsillectomy and tongue base reduction.

#### Severity index

The severity index is calculated by adding the digits representing the severity of obstruction at each anatomic structure/level [23]. For a patent airway with a score of N_0_V_0_Ts_0_O_0_Tb_0_E_0_L_0_ is 0. A single-level epiglottic obstruction N_0_V_0_Ts_0_O_0_Tb_0_E_2AP_L_0_ is 2. A multilevel obstruction at the tonsil and tongue base, N_0_V_0_Ts_2L_O_0_Tb_2AP_E_0_L_0_, has a severity index of 4.

### Relational mapping

#### Pringle et al. [15]

Grade 1 (G1_P_), simple palatal snoring consisted of the following properties: no obstruction (velum, AP, C, L, 0), noise production, vibration of soft palate (velum, AP, 1), vibration of the walls of the velopharyngeal sphincter (velum, AP, 1) and upper oropharynx (velum, L, 1), flapping of soft palate during inspiration (velum, AP, 1), prolapse of the uvula into the nasopharynx during expiration (velum, AP, 1), narrowing of the velopharyngeal area in the anteroposterior (velum, AP, 1) and circumferential direction (velum, C, 1) and tonsils bulging into the narrowing of the airway (tonsils, L, 0, 1). Grade 1 obstruction was mapped to uDISE model as:

- G1_P_ → {V_0–1, AP, L, C_} U {Ts_0–1, L_}.

Grade 2 (G2_P_), single-level palatal obstruction consisted of the following properties: obstructive episodes (velum, 2), mild-to-severe obstruction (velum, 2), obstruction only at velopharyngeal level (velum), anteroposterior (velum, AP), lateral (velum, AP), circumferential obstruction (velum, C). Grade 1 obstruction was mapped to uDISE model as:

- G2_P_ → {V_2, AP, L, C_} U {Ts_2, L,_}.

Grade 3 (G3_+P_), multi-segmental involvement—intermittent oro-hypopharyngeal collapse consisted of the following properties: obstruction at the velopharyngeal level and oro-hypopharyngeal level during inspiration (velum, oropharynx, AP, L, C 2). Due to the constraint of a required obstruction at the oropharynx, velum was excluded. Grade 3 (G3_+P_) was mapped to oropharynx obstruction with anteroposterior, lateral and concentric configuration as shown below:

- G3_+P_ → {O_2, AP, L, C_}.

Grade 4 (G4_+P_), sustained multi-segmental obstruction consisted of the following properties: obstruction at the velopharyngeal level and oro-hypopharyngeal level during inspiration and expiration (velum, oropharynx, AP, L, C 2). Due to the oro-hypopharyngeal constraint, velum was excluded. A+ sign was used to indicate multisegment obstruction. Grade 4 obstruction (G4_+P_) was mapped to oropharynx obstruction with anteroposterior, lateral and concentric configuration as shown below:

- G4_+P_ → {O_2, AP, L, C_}.

Grade 5 (G5_P_), tongue base-level obstruction consisted of the following properties: obstruction occurs at the level of tongue base with either the tongue base appearing to fall back onto the posterior pharyngeal wall (tongue base, 0–2, AP) or the posterior pharyngeal walls appearing to close down onto the tongue base (tongue base, 0–2, AP); epiglottis obscures the laryngeal inlet and obstructs the airway (epiglottis, 0–2, AP). Grade 5 (G5_p_) obstruction was mapped to tongue base and epiglottis obstruction in uDISE model as shown below:

- G5_p_ → {T_0–2, AP, L, C_: E_0–2, AP, L_}.

Pringle and Croft grading system accounts for inspiration and expiration for grade 3 and grade 4 which is not accounted for in uDISE model as it is not widely regarded as an important factor. Also, it is not clear how the Pringle and Croft grading system classifies single-level obstruction at the oropharynx level, nose and larynx.

#### Kezirian et al. [21]

VOTE classification system consists of four levels (velum, oropharynx, tongue base and epiglottis); three degrees of obstruction (0—none, 1—partial, and 2—complete), and three configurations of obstruction (anteroposterior, lateral, and concentric).

Obstruction at the velum level (V_k_) with three degrees of obstruction (0–2) and three obstruction configurations [anteroposterior (AP), lateral (L) and concentric (C)] in VOTE classification was directly mapped to obstruction at the velum level (V) with three degrees of obstruction (0–2) and three obstruction configurations [anteroposterior (AP), lateral (L) and concentric (C)] in uDISE model:

- V_k 0–2, AP, L, C_ → {V_0–2, AP, L, C_}.

Obstruction at the oropharynx level (O_k_) with three degrees of obstruction (0–2) and one obstruction configuration (L) in the VOTE classification system was mapped directly to the oropharynx (O) and tonsils with three degrees of obstruction (0–2) and one obstruction configuration (L) in the uDISE model:

- O_k 0–2, L_ → {O_0–2, L_} U {Ts_0–2, L_}.

Obstruction at the tongue base level (T_k_) with three degrees of obstruction (0–2) and obstruction configuration in AP direction in the VOTE classification system was mapped directly to tongue base level (Tb) with three degrees of obstruction (0–2) and obstruction configuration in AP direction in uDISE model:

- T_k 0–2, AP_ → {T_0–2, AP_}.

Obstruction at the epiglottis level (E_k_) with three degrees of obstruction (0–2) and two obstruction configurations in anteroposterior and lateral direction in the VOTE classification system was mapped directly with obstruction at the epiglottis level (E) with three degrees of obstruction (0–2) and two obstruction configurations in anteroposterior and lateral direction in uDISE model:

- E_k 0–2, AP, L_ → {E_0–2, AP, L_}.

VOTE classification does not consider obstruction at the nose and larynx [22]. In addition, VOTE does not account for concentric obstruction at the oropharynx level [33] and lateral and concentric obstruction at the tongue base [7].

#### Vicini et al. [22]

NOHL classification system consists of four levels [nose (N_v_), oropharynx (O_v_), hypopharynx (H_v_), and larynx (L_v_)]; three degrees of obstruction (0–25%: 1, 25–50%: 2, 50–75%: 3, 75–100%: 4) and three configurations of obstruction [anteroposterior, transversal (t), and concentric].

Obstruction at the nose was mapped as:

- N_V 1–4_ → {N_0–2_}.

Due to the static nature of obstruction at the nasal level, the configuration of the obstruction is not important. Furthermore, the effect of nasal obstruction on OSA is still debatable.

Obstruction in the transverse direction of the oropharynx was mapped as:

- O_V t, 1–2_ → {O_0, L_}; O_V t, 3_ → {O_1, L_}; O_V t, 4_ → {O_2, L_}.

Obstruction in the concentric direction of the oropharynx was mapped as:

- O_V C, 1–2_ → {O_0, C_}; O_V c, 3_ → {O_1, C_}; O_V c, 4_ → {O_2, C_}.

Palatine tonsillar hypertrophy grade (grade 3 or 4) was mapped as:

- Ts_V 3, 4_ → {Ts _2, L_}.

Obstruction in the AP direction of the tongue base was mapped as:

- H_V AP, 1–2_ → {T_0, AP_}; H_V AP, 3_ → {T_1, AP_}; H_V AP, 4_ → {T_2, AP_}.

Obstruction in the lateral direction of the tongue base was mapped as:

- H_V L, 1–2_ → {T_0, l_}; H_V L, 3_ → {T_1, L_}; H_V L, 4_ → {T_2, L_}.

Obstruction in the concentric direction of the tongue base was mapped as:

- H_V C, 1–2_ → {T_0, l_}; H_V C, 3_ → {T_1, L_}; H_V C, 4_ → {T_2, L_}.

Obstruction in the AP direction of the epiglottis was mapped as:

- L_N_ → {E_0, AP_}; L_P_ → {E_1–2, AP_}.

Obstruction in the lateral direction of the epiglottis was mapped as:

- L_N_ → {E_0, L_}; L_P_ → {E_1–2, L_}.

Anteroposterior, lateral and concentric obstruction at the velum in uDISE model was mapped as follows:

- V_0, AP_ → {O_V AP, 1–2_}; V_1, AP_ → {O_V AP, 3_}; V_2, AP_ → {O_V AP, 4_},
- V_0, L_ → {O_V t, 1–2_}; V_1, L_ → {O_V t, 3_}; V_2, L_ → {O_V t, 4_},
- V_0, C_ → {O_V C, 1–2_}; V_1, C_ → {O_V C, 3_}; V_2, L_ → {O_V C, 4_}.

#### Veer [29]

P-T-L-Tb-E classification system evaluated obstruction at five key anatomical sites: palate (P_V_), tonsils (T_V_), lateral wall (L_V_), tongue base (Tb_V_) and epiglottis (E_V_), three degrees of obstruction (0—none, <50%—partial, and >50%—complete) and three configurations of obstruction (anteroposterior, lateral, and concentric).

Obstruction at the palate level (P_V_) with anteroposterior and concentric obstruction was mapped to the obstruction at the velum (V) with anteroposterior and concentric obstruction:

- P_V 1_ → {V_0, AP, C_}; P_V 2_ → {V_1–2, AP_}; P_V 3_ → {V_1–2, C_}.

Obstruction at the tonsils was mapped as obstruction at the tonsil level with lateral obstruction configuration:

- T_V 1_ → {Ts_0, L_}; T_V 2_ → {Ts_1, L_}; T_V 3_ → {Ts_2, L_}.

Obstruction at the lateral pharyngeal wall was mapped to obstruction at the oropharynx with lateral obstruction configuration:

- L_V 1_ → {O_0, L_}; L_V 2_ → {O_1, L_}; L_V 3_ → {O_2, L_}.

Obstruction at the tongue base was mapped to obstruction at the tongue base with anteroposterior obstruction configuration:

- Tb_V 1_ → {T_0, AP_}; Tb_V 2_ → {T_1, AP_}; Tb_V 3_ → {T_2, AP_}.

Obstruction at the epiglottis was mapped to obstruction at the epiglottis with anteroposterior obstruction configuration:

- E_V 1_ → {E_0, AP_}; E_V 2_ → {E_1–2, AP_}.

It is worth mentioning that obstruction at the nose and larynx are not considered in P-T-L-Tb-E classification system. Furthermore, obstruction in the lateral direction at the velum level, obstruction at the oropharynx in the anteroposterior and concentric direction, obstruction at the tongue base in the lateral and concentric direction and obstruction at the epiglottis in the lateral direction is not considered in P-T-L-Tb-E classification system.

### Limitations

This study has its three key limitations. First, uDISE model is a model based on an amalgamation of the strength of existing DISE classification system which inherently limits the scope of adding new anatomical structures and configurations of obstructions. Second, uDISE model was related to four existing DISE classification systems. More research is needed to relate uDISE model with other existing DISE classification systems. Third, a randomised clinical study is required to validate the accuracy and reliability of uDISE model.

## Conclusion

First, uDISE model is a new DISE classification system for analysing visual findings based on DISE in an easy, accurate, objective and systematic way. Second, uDISE model provides a framework for mapping different DISE findings across different classification systems into one common DISE assessment format. Hence, this provides a methodology for comparing different DISE assessment, treatment plan and treatment outcome irrespective of initial DISE classification system used. Third, uDISE model provides a frame work to relate assessment score with treatment plan and potentially predicts treatment outcome. Fourth, uDISE model provides both a human and computational framework for systematically classifying DISE findings. Further research is needed to clinically validate uDISE model.
